# Effect of Local Basic Fibroblast Growth Factor and Vascular Endothelial Growth Factor on Subcutaneously Allotransplanted Ovarian Tissue in Ovariectomized Mice

**DOI:** 10.1371/journal.pone.0134035

**Published:** 2015-07-24

**Authors:** Jiangman Gao, Ying Huang, Min Li, Hongcui Zhao, Yue Zhao, Rong Li, Jie Yan, Yang Yu, Jie Qiao

**Affiliations:** 1 Center of Reproductive Medicine, Department of Obstetrics and Gynecology, Peking University Third Hospital, Beijing, 100191, China; 2 Key Laboratory of Assisted Reproduction, Ministry of Education, Beijing, 100191, China; 3 Beijing Key Laboratory of Reproductive Endocrinology and Assisted Reproductive Technology, Beijing, 100191, China; China Agricultural University, CHINA

## Abstract

**Objective:**

One of the major obstacles to ovarian tissue preservation is delayed angiogenesis that leads follicles lost after transplantation. The aim of the present study was to investigate the effects of bFGF and VEGF on heterotopic transplanted ovarian tissue using a mouse model.

**Methods:**

Female mice underwent bilateral ovariectomy. Ovarian tissues encapsulated by fibrin hydrogels were transplanted subcutaneously into recipient mice, in which ovarian hormonal cyclicity was absent. The fibrinogen solution was mixed with bFGF, VEGF, or a mixture of bFGF and VEGF. The grafts were recovered 21 days after transplantation. Follicle morphology and follicle numbers were observed by H&E staining. Blood vessels were observed in transplanted intra-ovarian tissue by CD31 antibody IHC staining. Daily vaginal cytology was performed to determine estrous cycle and functional restoration of transplanted ovarian tissue. Blood was collected weekly and serum FSH levels were measured with a radioimmunoassay kit. Apoptosis analysis was performed by anti-AC-3 staining and survivin mRNA expression.

**Results:**

The number of primordial follicles and secondary follicles in the bFGF+VEGF group was significantly higher than in the control group. The vascular density in the bFGF+VEGF groups were significantly higher than in the bFGF and the VEGF groups; there was no significant difference between the bFGF and VEGF groups. Estrous cycle was earlier in the bFGF+VEGF group compared with the control group; all mice in this group restored ovarian function. Serum FSH levels in the bFGF+VEGF group were significantly lower than in the control group by day 14 post-transplantation. The AC-3-positive in control group was significantly higher compared with bFGF group and VEGF group, and in bFGF+VEGF group was significantly lower than bFGF group and VEGF group. Survivin mRNA expression in bFGF+VEGF group was significantly higher than control group.

**Conclusion:**

The combination of bFGF and VEGF has beneficial effects on follicle survival, angiogenesis, and resumption of estrous cycles.

## Introduction

In women, approximately one tenth of cancers occur in <45 years old. More than 90% of girls and young women with cancer require chemotherapy, radiotherapy, or bone marrow transplantation for curative treatment[1]. However, the gonadotoxicity of ionizing radiation and chemotherapeutic drugs can frequently lead to premature ovarian failure (POF) and loss of endocrine and reproductive function, conditions with serious long-term hormone-related consequences and infertility. The severity of these effects depends on follicular reserve, patient age, and the type and dose of drugs used.

Thus, fertility preservation for female cancer patients is a major concern, and can offer an optimal quality of life to these young cancer survivors [2]. Compared with the cryopreservation of oocytes and embryos, ovarian tissue cryopreservation requires neither a sperm donor nor ovarian stimulation, which would be appropriate for women who require immediate cancer treatment or who have contraindications for ovarian stimulation. Since 2004, there have been 37 live births after orthotopic retransplantation of frozen/thawed ovarian tissue[3]. The overall pregnancy rate after transplantation of ovarian tissue has been estimated to be between 11% and 30% [2].

Successful pregnancy is great but not the only goal for these patients and doctors. The other advantage of transplantation of frozen/thawed ovarian tissue is to restore the steroidogenic function of ovaries so as to alleviate menopausal symptoms. For these patients, ovarian tissue is implanted into the subcutaneous tissue instand of ovarian orthotopic transplantation, as opposed to laparotomy, which carries more surgical risk and financial cost. However, the return of endocrine function is debatable and the longevity of transplanted ovarian function is expected to be relatively short, likely due to the rate of follicular loss after ovarian transplantation [4]. Numerous studies have shown that hypoxia due to delayed revascularization is associated with massive primordial follicle loss and limits the longevity and success of ovarian transplantation [5–7]. Thus, strategies to improve and hasten graft vascularization and reduce follicular loss are needed.

The regulation of angiogenesis is a complex process that involves multiple vasoactive and angiogenic factors [8]. Vascular endothelial growth factor (VEGF) and basic fibroblast growth factor (bFGF) play important roles in angiogenesis. VEGF is an endothelial cell-specific mitogen *in vitro* and an angiogenic inducer in a variety of *in vivo* models. Signal transduction involves binding to tyrosine kinase receptors and results in endothelial cell proliferation, migration, and neovascularization [9, 10]. bFGF is of the FGF family, and plays important roles in various developmental processes, such as stimulating endothelial cell migration and mitosis, and maintaining granulosa cell viability during follicular development. It is a potent and effective angiogenic inducer. Several studies have indicated that bFGF and VEGF have synergistic effect in the angiogenic process [11, 12]. In this study, we investigated the effects of bFGF, VEGF, or the combination on heterotopic transplanted ovarian tissue using a mouse model, by assessing follicle number, vascular reconstruction, apoptosis, estrous cycle, and the level of serum follicular stimulating hormone (FSH).

## Materials and Methods

Use of animal and tissue for this study, and the study itself were approved by the Institutional Review Board at Peking University Third Hospital. All chemicals used in this study were purchased from Sigma-Aldrich Chemical Co. (St. Louis, MO, USA) unless otherwise stated.

### Mice

All animal experiments were approved by the Institutional Animal Care and Use Committee of Peking University Third Hospital, China. ICR outbred mice (CD-1; Vital River Laboratories) were housed in the Animal Center of Medical College of Peking University, under controlled temperature and lighting conditions, according to national legislation for animal care, and given food and water *ad libitum*.

Six-week-old female mice were anesthetized with an intraperitoneal injection of 2,2,2-trobromoethyl alcohol and tertamyl alcohol, followed by bilateral ovariectomy in sterile conditions. Vaginal cytology was performed to verify the absence of ovarian function after ovariectomy. These mice received ovarian tissue transplants 2 weeks after ovariectomy.

### Ovarian tissue collection and treatment

Ovarian tissues were isolated from 18-day-old ICR mice, collected and immersed in Leibovitz-15 medium supplemented with 100 mg/ml of streptomycin and 100 IU/ml of penicillin at room temperature. The ovaries were divided into two parts using sterile blades.

The fibrinogen solution (80 mg/ml) was made with lyophilized fibrinogen from human plasma (87% clot table protein) in 0.9% saline at 37°C. The fibrinogen solution was mixed with bFGF (Gibco), VEGF (PMG0111, Invitrogen), or a mixture of bFGF and VEGF. The concentration of bFGF and VEGF is 100μg/ml and 75μg/ml respectively. We placed 5μl fibrinogen solution droplets on a hydrophobic surface, transferred ovarian tissue into the fibrinogen droplets and added 2.5 μl of thrombin solution (prepared in a 0.1% (w/v) BSA solution) to cross-link for 5 min. Ovarian tissue grafted with only fibrin hydrogels was assigned to the control group.

### Heterotopic transplantation and graft recovery

Encapsulated ovarian tissue was transplanted subcutaneously into the posterior necks of recipient mice, in which ovarian hormonal cyclicity was absent. Daily vaginal cytology was performed to determine the onset of hormonal cyclicity (grafted ovarian tissue function); weekly blood draws were performed from the inner canthus to determine serum FSH levels. FSH levels were measured with radioimmunoassay kits (Beijing North Institute of Biological Technology, Beijing, China).

### Histologic analysis and immunohistochemistry

Animals were anesthetized as described above and the grafts were recovered and fixed in 4% formaldehyde, 21 days after transplantation. The transplanted grafts were prepared for paraffin embedding and hematoxylin and eosin (HE) staining. Parameters of histologic evaluation included follicular density, as well as tissue and oocyte general morphology. Follicles were counted in a 100-fold field in 10 different sections, when the dark staining nucleolus was visualized within the nucleus of the follicles. The average number of primordial, primary, and secondary follicles was calculated in each section.

Recovered grafts underwent immunohistochemistry (IHC) staining, for CD31, to assess blood vessel density in ovarian tissue. CD31 antibody (Abcam, Cambridge, U.K.) was used to identify epithelial cells of new blood vessels. Anti-AC-3 staining evaluated apoptosis in ovarian tissues on day 7 after transplantation. These procedures have been described previously[13]. Briefly, sections were deparaffinated, rehydrated, demasked, and endogenous peroxidase activity was blocked by incubation in 0.3% H_2_O_2_ for 10 min. Sections were incubated with primary antibodies overnight and subsequently incubated for 30 min at room temperature with secondary antibodies. DAB was added to change the color and counterstaining was done with haematoxylin.

AC-3 staining levels were graded according to intensity and quantity: 0 = no apoptosis; 1 = very little apoptotic cells with low staining intensity; 2 = few apoptotic cells and medium staining intensity; and 3 = many apoptotic cells and high staining intensity.

### Assessment of survivin mRNA expression by RT-PCR

Total RNA was isolated by TRIzol reagent (Invitrogen Life Technologies, Carlsbad, CA). The cDNA was produced with 1 mg total RNA using the cDNA Synthesis Kit (Thermo Fisher, Marietta, OH, USA), according to the manufacturer’s instructions. The oligonucleotide sequences of the survivin primers used were 5'-ATCGCCACCTTCAAGAACTG-3' for sense and 5'-TGACTGACGGGTAGTCTTTGC-3' for antisense. For quantitative polymerase chain reaction (PCR), amplification was carried out in an ABI7500 (Applied Biosystems, Foster City, CA) using the SYBR Green Kit (Applied Biosystems) as follows: 95°C for 2 min, then 40 cycles of denaturation at 95°C for 30 s, 60°C for 1 min, and annealing and elongation at 72°C for 1 minute each,. All samples were studied in triplicate. GAPDH was chosen as the internal control for gene expression normalization. The relative expression level of all target genes were calculated using the 2exp-ΔΔCT method.

### Statistical analysis

Statistical analysis was performed with IBM SPSS statistical software, version 20.0 (Chicago, IL, USA). All data in this study was analyzed by one-way ANOVA followed by an LSD post hoc test for multiple comparisons, unless otherwise stated. The time of cyclicity resumption and serum FSH levels were compared using the rank-sum test at different time points, compared with starting levels. A p-value of less than 0.05 was considered to statistically significant.

## Results

### Impact on follicle survival

**Fig 1:**
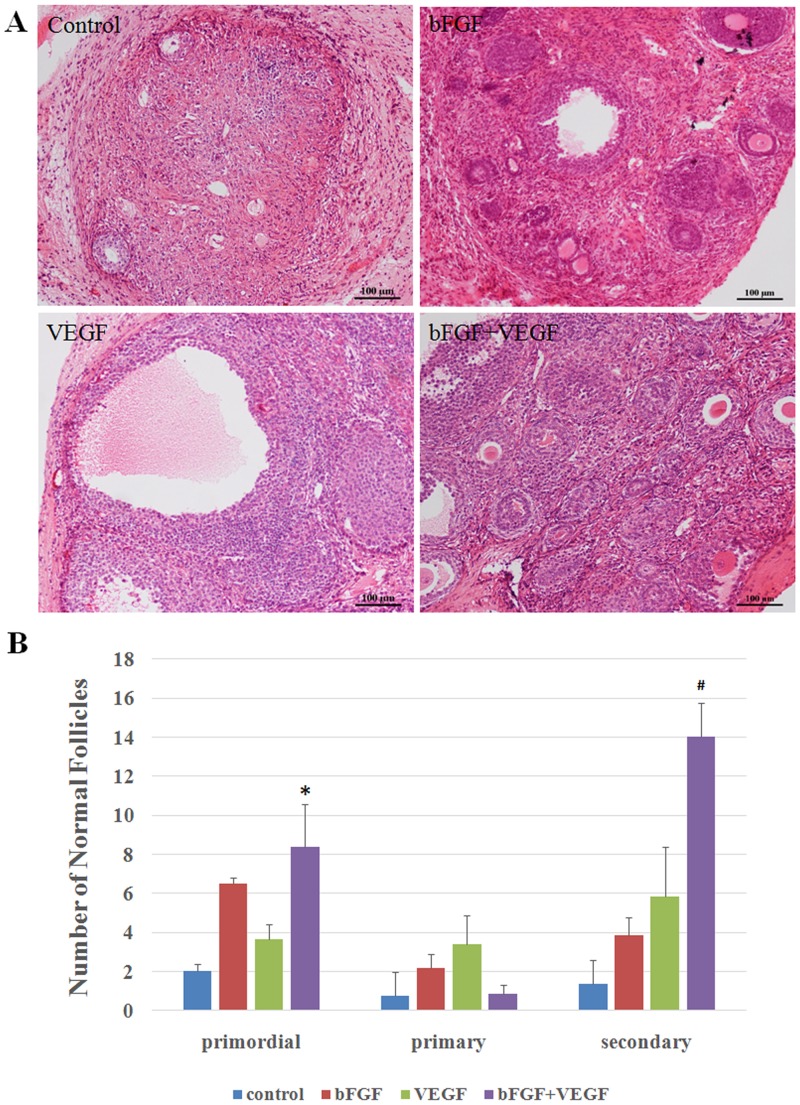
Ovarian tissue morphology after transplantation. **(A)** Representative micrograph of H&E-stained ovarian sections showing the preserved follicles. Original magnification100×. Scale bar = 100 μm. **(B)** The quantity of normal follicles at different developmental stages, including primordial, primary, and secondary follicles. * and # indicate that the p-value was <0.05 compared with the control group for primordial and secondary follicles, respectively.

After 3 weeks, all grafts were retrieved, and most were found to be bigger than their original tissue size. There were even large antral follicles at the periphery of ovarian tissue grafts, indicating that the follicles had grown after transplantation and that ovulation would possibly occur soon (Fig 1A). The number of primordial and secondary follicles in the bFGF group and in the VEGF group was higher than that in the control group, but no significantly difference were found. The number of primordial and secondary follicles in the bFGF+VEGF group was significantly higher than in the control group, and was higher than in the bFGF and VEGF groups though there was no significant difference (Fig 1B). There were no differences among the four groups with regards to the number of primary follicles.

### Vascularization of ovarian grafts after transplantation

**Fig 2:**
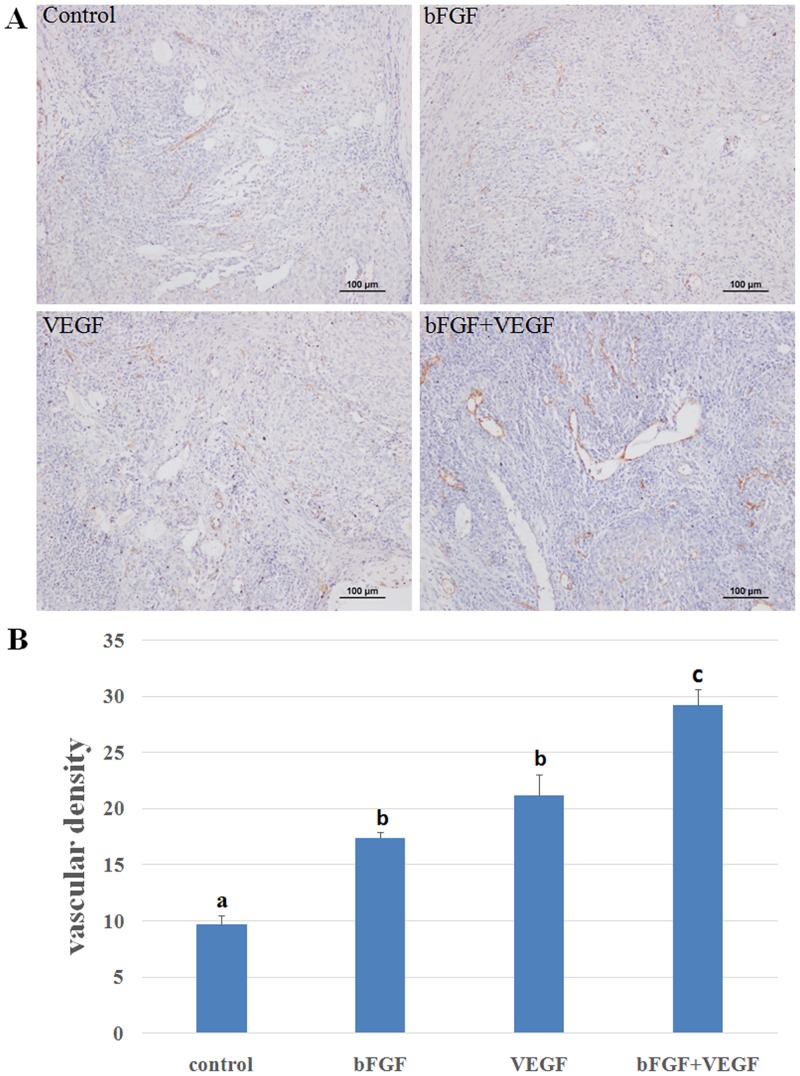
Ovarian section stained for CD31 expression. **(A)** Red-brown coloring was specified as positive staining. Original magnification100×. Scale bar = 100 μm. **(B)** The vascular density was measured by immunohistochemical staining for CD31 in the transplanted ovarian tissues. A significant difference existed among a, b, and c. (p<0.05).

Blood vessels were evidenced in transplanted intra-ovarian tissue by CD31 antibody IHC staining (Fig 2A). We found that CD31 expression was significantly higher in groups with added angiogenetic factors, compared with controls. The vascular density in the bFGF+VEGF group was significantly higher than in the bFGF and VEGF groups. There was no significant difference found with regards to vascularization between the bFGF and VEGF groups, although the number of blood vessels in the VEGF group was higher than in the bFGF group (Fig 2B).

### Ovarian function restored in mice

**Fig 3:**
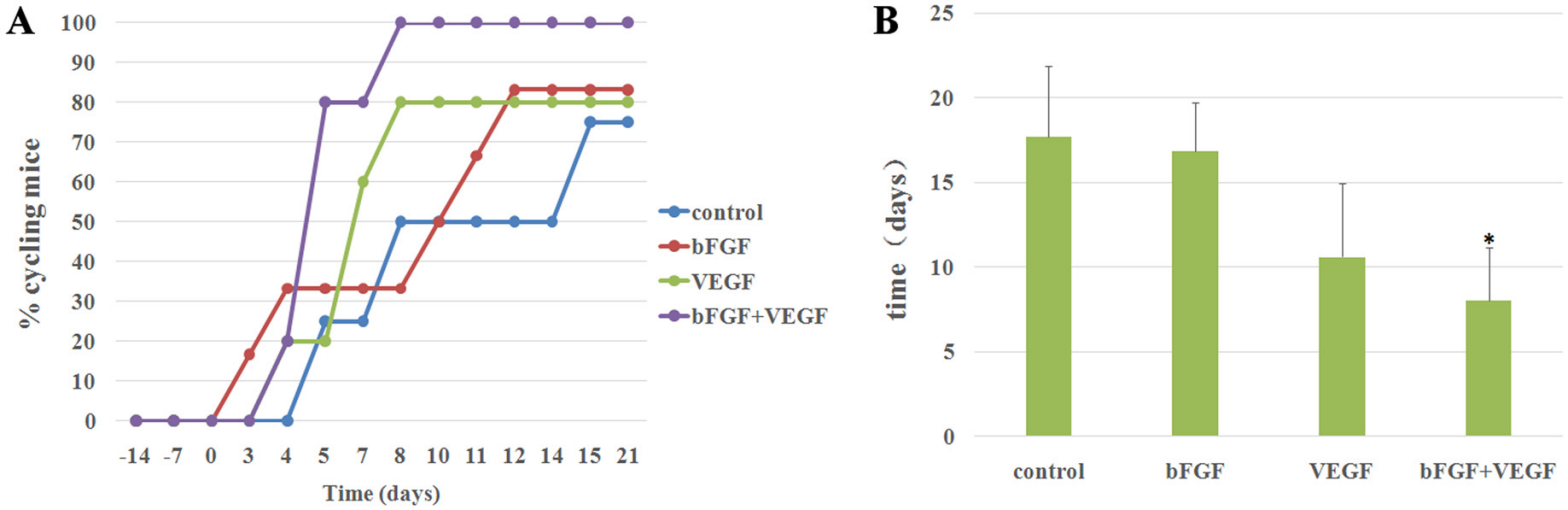
Restoration of ovarian function after transplantation. **(A)** Restoration of estrous cycle after transplantation, presented as plots. Time point 0 indicates that the animals were transplanted with ovarian tissue; the percentage of cycling animals is shown on the y axis, corresponding to different time points. **(B)** The average time of restoration of ovarian function after transplantation. *indicates that the p-value was <0.05, compared with the control group.

Hormonal cycling ceased after bilateral ovariectomy, which was confirmed by vaginal cytology. Daily performance of vaginal cytology determined the degree of restoration of transplanted ovarian tissue function. When grafts recovered 3 weeks after transplantation, most of the mouse hosts recovered hormonal cyclicity. Based on cytology results, mice transplanted with bFGF, VEGF, or bFGF+VEGF resumed cyclicity earlier than in the control group. This was especially true in the bFGF+VEGF group and all mice in bFGF+VEGF group restored ovarian function (Fig 3).

We also investigated FSH levels at different experimental times (Table 1). FSH serum levels remained elevated one and two weeks after ovariectomy. Serum FSH levels decreased significantly 2 weeks post-transplantation and were even undetectable 3 weeks post-transplantation. Declining and undetectable serum FSH levels indicated that grafted ovarian tissue had begun to function. Daily vaginal cytology was consistent with FSH data. Serum FSH levels in the bFGF+VEGF group was lower than other groups 7 days after transplantation, and was significantly lower than in the control group 14 days after transplantation.

**Table 1:** FSH levels were determined once a week.

Groups	Levels of FSH (mIU/ml)					
	Day -14	Day -7	Day 0	Day 7	Day 14	Day 21
**Control**	<0.01	6.31 ±0.33	16.81 ±1.12	13.13 ±0.72	3.55 ±0.32	2.16 ±0.54
**bFGF**	<0.01	5.86 ±0.21	15.59 ±0.8	10.45 ±0.89	1.34 ±0.11	0.56 ±0.04
**VEGF**	<0.01	5.79 ±0.60	18.92 ±2.15	11.02 ±1.24	1.09 ±0.09	<0.01
**bFGF+VEGF**	<0.01	6.11 ±0.42	15.76 ±1.47	8.42 ±0.82	0.33 ±0.04*	<0.01

Day -14 corresponds to ovariectomy and day 0 indicates the transplantation time points.

* Statistically significant difference compared with control group on the day 14 post-transplantation, P <0.05.

### Apoptosis analysis

**Fig 4:**
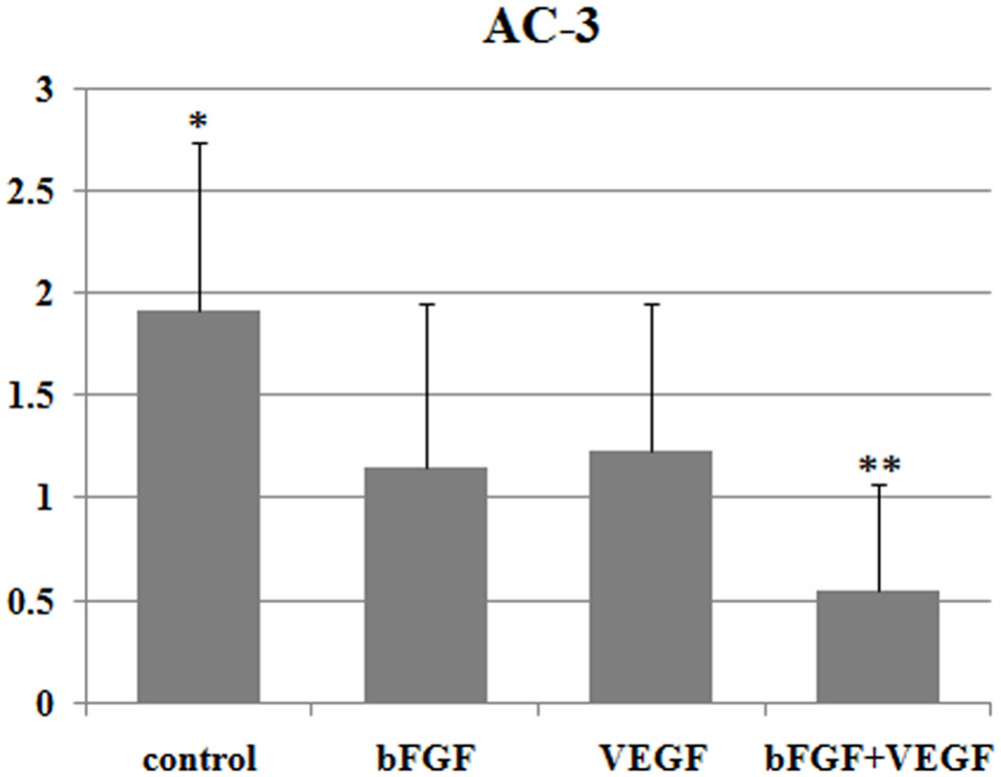
Apoptosis expression in grafts 7 days after grafting. Results are presented as mean ± SD. Apoptosis (AC-3) levels were graded according to intensity and quantity: 0 = no apoptosis; 1 = few apoptotic cells with low staining intensity; 2 = apoptotic cells and medium staining intensity; and 3 = many apoptotic cells and high staining intensity.*Significantly higher than bFGF treatment and VEGF treatment (P <0.05). **Significantly lower than bFGF treatment and VEGF treatment (P<0.05).

**Fig 5:**
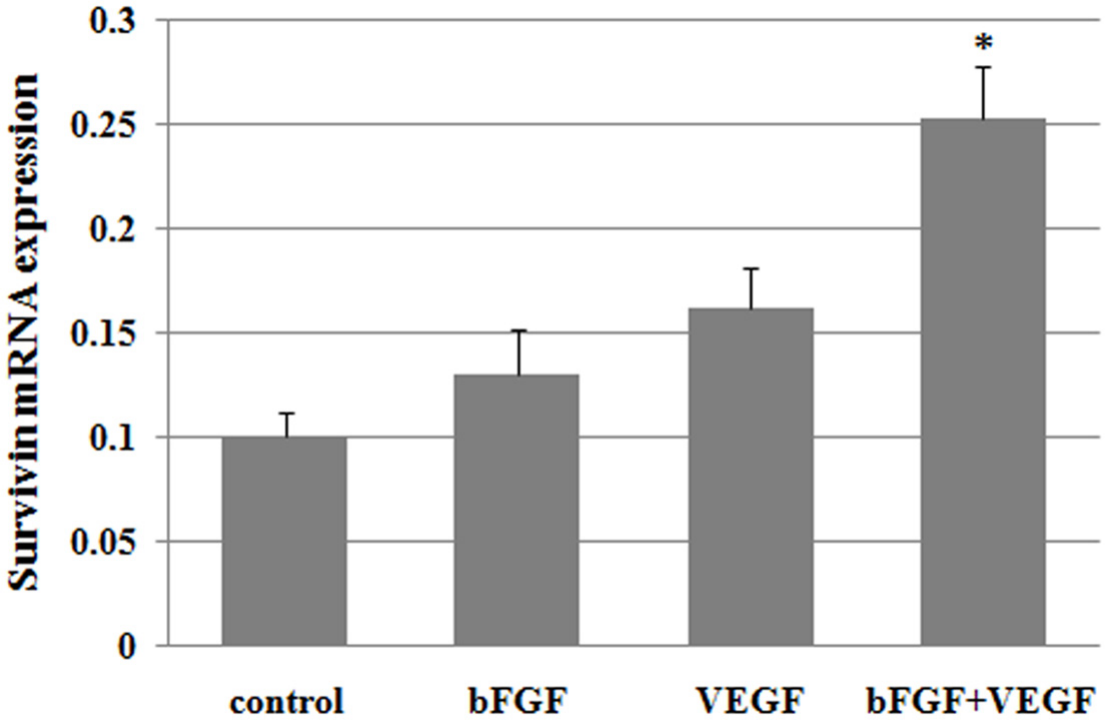
Survivin mRNA expression in grafts 21 days after grafting. *P<0.05 significantly higher than control group.

Grafts were immunostained with anti-AC-3 staining to evaluate apoptosis in ovarian tissues on Day 7 after transplantation. The AC-3-positive in control group was significantly higher compared with in the bFGF and VEGF group (P<0.05), and AC-3-positive in bFGF+VEGF group was significantly lower than the bFGF and VEGF group (P<0.05) (Fig 4). Survivin is a member of the inhibitors of apoptosis protein family which has important role in apoptotic resistance, cell growth, angiogenesis and follicular development, involved in both control of apoptosis and regulation of cell division during mitosis. We performed qRT-PCR analysis to detect survivin mRNA levels in ovarian tissues on day 21 after transplantation. Survivin mRNA expression in bFGF+VEGF group was significantly higher than control group (Fig 5).

## Discussion

Massive loss of follicles after the ovarian transplantation limits the success of the ovarian tissue cryopreservation and transplantation techniques. Hypoxia in the transplant from delayed revascularization is the major cause of premature follicle depletion[14]. Therefore, there is a great clinical need to enhance neoangiogenesis after ovarian transplantation and to improve the clinical utility of this procedure. bFGF and VEGF play important roles in angiogenesis and have been widely used. This study was therefore designed to investigate whether bFGF, VEGF, or the combination could improve the quality of heterotopic allotransplanted ovarian tissue using a murine model. We found that bFGF and VEGF have beneficial effects on follicle survival, angiogenesis, and resumption of the estrous cycle, especially when they are used together.

Survival and normal development of follicle is a prerequisite after ovarian tissue transplant, which adjusts endocrine function and provides fertility potency. In previous studies, it has been shown that bFGF and VEGF can promote follicle development, which has been indicated for both *in vitro* or *in vivo* conditions. Under *in vitro* conditions, VEGF stimulated pre-antral follicle growth in bovine and rat models [15, 16]. bFGF also had the advantageous role of promoting early follicle growth in the mouse, goat, and human. The function of VEGF and bFGF in promoting follicle development was attributed to regulating cytokinetic interaction. In the *in vivo* model, direct injection of VEGF into the ovary of mice promotes follicular development and diminishes apoptosis [17]; local inhibition of VEGF activity increases apoptosis in ovaries, leading a larger number of follicles to atresia [18]. It was fund that bFGF was essential for follicle activation and development, and supplementation with bFGF could promote the development of human ovarian early follicles during growth in vitro[19]. We also suggested that bFGF was beneficial for follicle survival and growth after transplantation in our previous study [13]. In this study, we found that at different developmental stages, follicle numbers were increased as long as either bFGF or VEGF were applied; however, a more significant difference was found when bFGF and VEGF were combined.

Efficient revascularization after tissue grafting contributed to the resumption of ovarian function and the longevity of the graft [7]. It has been suggested that bFGF and VEGF play important roles and may act synergistically during the process of angiogenesis *in vitro* and *in vivo*. bFGF increased VEGF expression in the endothelial cells(ECs) and several other types of cells [12, 20, 21]; the role of bFGF to enhance angiogenesis was significantly inhibited by VEGF suppression, as when VEGF-neutralizing antibody was used [22]. bFGF also induced the expression of VEGF receptor KDR through protein kinase C and the p44/p42 mitogen-activated protein kinase-dependent pathway [23]; VEGF receptor antagonists inhibited bFGF-induced angiogenesis *in vivo* and *in vitro* [24]. In addition, the capacity of VEGF, which induces u-PA-plasmin activity and angiogenesis, depended on endogenous bFGF produced by ECs [25]. Combined administration of bFGF and VEGF showed a synergistic effect to enhance angiogenesis of spinal cord bridges following injury and osteogenic differentiation of rat osteoblasts [26, 27]. In the *in vitro* angiogenesis model, both bFGF and VEGF demonstrated angiogenic effects on human microvascular endothelial cells in a dose-dependent manner; when combined, these two factors also acted synergistically [11].

Therefore, in addition to promoting follicle development, the other role of bFGF and VEGF to improve angiogenesis in ovarian transplantation has been found. Improved expression of VEGF was found in grafted ovaries two days after transplantation [28], which suggested its important role in angiogenesis. Similarly, bFGF also significantly improved angiogenesis, while apoptosis of follicles and stromal cells was significantly decreased in grafted ovaries seven days after transplantation [13]. In our study, we also found a similar promotion of angiogenesis in transplanted ovarian tissues, with the bFGF and VEGF combination displaying superior effects.

A close relationship has been found between angiogenesis and survival of transplanted samples in the clinic. For ovarian tissue transplant, its survival determined whether endocrine function could be recovered and maintained in the long term. VEGF delivery may improve survival of transplanted human ovarian tissue [5, 29] by accelerating blood vessel recruitment and functional angiogenesis [30]; a similar role was implied when bFGF was applied [13]. Moreover, Wang et al. [31] xenotransplanted fresh human ovarian tissue into rabbits, and suggested that the combined administration of VEGF and bFGF may improve angiogenesis, reduce apoptosis and fibrosis, and increase survival in xenotransplanted human ovarian tissue, which was in accordance with our results.

However, a critical aim of heterotopic ovarian transplantation is restoration of ovarian function, in order to regulate endocrine disorders related to ovarian steroid hormones. In our study, we proved that the estrous cycle can be recovered in a shorter period of time; FSH levels, one of most important gonadotropins depending on a hormone feedback from the ovary, were also decreased. Effective and timely angiogenesis after transplantation is critical for the survival and functional restoration of ovarian grafts. We have shown that bFGF and VEGF improves angiogenesis in grafted ovarian tissues. In addition, bFGF and VEGF are involved in the transition and development of the follicle. It has been reported that both bFGF and VEGF have effects on E_2_ and progesterone production of cultured granulosa cells [32]. The fluctuation of E_2_ and progesterone directly determines the timing of the menstrual cycle. It is probably for the above reason that, in our study, mice with transplanted ovarian tissue treated by angiogenic cytokines resumed estrous cycle earlier than the control group. Thus, the administration of VEGF and bFGF, especially the combination, may facilitate angiogenesis, promote follicle development, and trigger E_2_ and progesterone production, resulting to the cyclicity of vaginal cytology resumed earlier than the control group.

In conclusion, we investigated the roles of bFGF and VEGF during the process of mouse ovarian tissue transplantation, and demonstrated that the combination of bFGF and VEGF has beneficial effects on follicle survival, angiogenesis, and resumption of the estrous cycle. However, further investigations are necessary to elucidate the functional mechanism of VEGF and bFGF, in order to ensure the efficacy and safety of exogenous intervention for ovarian tissue transplant in humans.
